# Exposure to Bisphenol A Substitutes, Bisphenol S and Bisphenol F, and Its Association with Developing Obesity and Diabetes Mellitus: A Narrative Review

**DOI:** 10.3390/ijerph192315918

**Published:** 2022-11-29

**Authors:** Hend F. Alharbi, Raya Algonaiman, Rana Alduwayghiri, Thamer Aljutaily, Reham M. Algheshairy, Abdulkarim S. Almutairi, Razan M. Alharbi, Leena A. Alfurayh, Amjad A. Alshahwan, Amjad F. Alsadun, Hassan Barakat

**Affiliations:** 1 Department of Food Science and Human Nutrition, College of Agriculture and Veterinary Medicine, Qassim University, Buraydah 51452, Saudi Arabia; 2 Department of Food Technology, Faculty of Agriculture, Benha University, Moshtohor 13736, Egypt

**Keywords:** bisphenols, bisphenol S, bisphenol F, endocrine disruptors, obesity, diabetes

## Abstract

Bisphenol A, a well-known endocrine-disrupting chemical, has been replaced with its analogs bisphenol S (BPS) and bisphenol F (BPF) over the last decade due to health concerns. BPS and BPF are present in relatively high concentrations in different products, such as food products, personal care products, and sales receipts. Both BPS and BPF have similar structural and chemical properties to BPA; therefore, considerable scientific efforts have investigated the safety of their exposure. In this review, we summarize the findings of relevant epidemiological studies investigating the association between urinary concentrations of BPS and/or BPF with the incidence of obesity or diabetes. The results showed that BPS and BPF were detected in many urinary samples at median concentrations ranging from 0.03 to 0.4 µg·L^−1^. At this exposure level, BPS median urinary concentrations (0.4 µg·L^−1^) were associated with the development of obesity. At a lower exposure level (0.1–0.03 µg·L^−1^), two studies showed an association with developing diabetes. For BPF exposure, only one study showed an association with obesity. However, most of the reported studies only assessed BPS exposure levels. Furthermore, we also summarize the findings of experimental studies *in vivo* and *in vitro* regarding our aim; results support the possible obesogenic effects/metabolic disorders mediated by BPS and/or BPF exposure. Unexpectedly, BPS may promote worse obesogenic effects than BPA. In addition, the possible mode of action underlying the obesogenic effects of BPS might be attributed to various pathophysiological mechanisms, including estrogenic or androgenic activities, alterations in the gene expression of critical adipogenesis-related markers, and induction of oxidative stress and an inflammatory state. Furthermore, susceptibility to the adverse effects of BPS may be altered by sex differences according to the results of both epidemiological and experimental studies. However, the possible mode of action underlying these sex differences is still unclear. In conclusion, exposure to BPS or BPF may promote the development of obesity and diabetes. Future approaches are highly needed to assess the safety of BPS and BPF regarding their potential effects in promoting metabolic disturbances. Other studies in different populations and settings are highly suggested.

## 1. Introduction

Obesity and diabetes are two of the most important public health issues facing the world today. Together, they can be considered a global twin epidemic that is growing each year. Obesity was responsible for more than four million deaths in 2017 [1], while diabetes was responsible for more than six million deaths in 2021 [2]. By 2030, it is projected that the influence of obesity will not decline, whereas the prevalence of diabetes is expected to increase [3]. Pathophysiologically, obesity and diabetes can be attributed to insulin resistance and insulin deficiency, mainly due to the complex interactions between genetics and environmental factors such as energy-dense diets, sedentary lifestyles, or aging [4,5]. However, growing evidence has shown that manufactured environmental chemicals, mostly endocrine-disrupting chemicals (EDCs), are an additional risk factor that should be considered [6].

**Figure 1:**
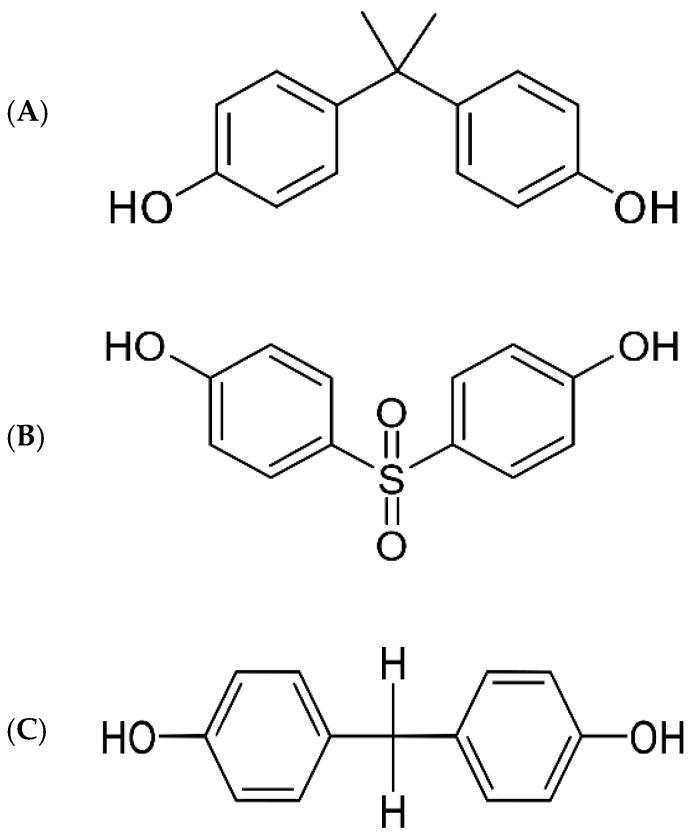
Chemical structures of bisphenol A (**A**), bisphenol S (**B**), and bisphenol F (**C**).

EDCs such as bisphenol A (BPA, 2,2-bis[4-hydroxyphenyl]propane) are synthetic chemicals capable of altering or interacting with the body’s endocrine system; they have been widely used for decades in the lining of canned food and drinks, packaging of baby formula and baby bottles, dental implants, and sales receipts. EDCs can leak monomers into food and beverages, thus disrupting endocrine pathways by mimicking or blocking hormones when absorbed into the body. BPA has been well demonstrated to mimic estrogen by binding to its receptors, leading to obesogenic effects and metabolic disorders [7,8]. In several *in vivo* and *in vitro* studies, BPA exposure was shown to promote significant disturbances in glucose homeostasis and insulin sensitivity. Impairments in lipid metabolism and the promotion of fat accumulation were also observed [9,10,11]. In addition, epidemiological studies have reported that urinary and/or serum BPA concentrations are strongly associated with an increased incidence of obesity [12,13,14] and diabetes [15,16,17,18]. Therefore, BPA has been banned in many countries due to the growing evidence of its potential adverse effects [19]. In 2012, the US Food and Drug Administration (FDA) clarified a statement regarding abandoning the use of BPA in baby products [20]. Since then, BPA-free products have rapidly increased on the market, although these have been manufactured by replacing BPA with its analogs, such as bisphenol S (BPS, 4,4′-sulfonyldiphenol) and bisphenol F (BPF, 4,4′-dihydroxydiphenylmethane) (see Figure 1).

Over the last decade, BPS and BPF have been widely used by manufacturers as a substitute for BPA; they are present in a wide range of products such as food products, cleaning agents, thermal papers, dental sealants, and personal care products [21]. High concentrations of BPF were found in different vegetable and seafood products in China [22]. In thermal paper receipt samples, BPS was detected in 62% of samples from Italy [23] and all samples from the US, Japan, Korea, and Vietnam [24]. Furthermore, BPS and BPF were detected in 89.4% and 66.5% of urinary samples from US adults and children, respectively [25]. In seven Asian countries, BPS tests revealed positive results in 81% of the population [26]. Therefore, as a large population is exposed to BPA substitutes at a relatively high level, the safety of BPA substitutes has been questioned over the last few years.

Indeed, BPS and BPF are structurally and chemically similar to BPA (Figure 1); thus, they are expected to promote adverse effects by acting as endocrine disrupters [11]. Recently, BPS and BPF were reported to promote fat accumulation in adipocytes [27,28]. Disturbances in glucose and insulin homeostasis were also reported after BPS treatment in rodents [29]. In addition, BPS might promote obesogenic effects in a worse manner compared to BPA [8]. This review aimed to summarize the findings of epidemiological and experimental studies providing evidence on the potential obesogenic and diabetic effects of BPS and BPF exposure.

## 2. Urinary Concentrations of BPS/BPF and the Incidence of Obesity and Diabetes

Exposure to BPA substitutes, BPS and BPF, has been detected in urinary samples of human participants in multiple countries (Table 1); the association between urinary concentrations of BPS and/or BPF and developing obesity and diabetes has been studied. This section discusses the findings of these studies.

### 2.1. Incidence of Obesity

In a representative US sample including more than 1500 adults aged ≥20 years from the National Health and Nutrition Examination Survey (NHANES) (2013–2014) [30], results showed that obese participants had higher median urinary concentrations (MUC) of three bisphenols, BPA, BPS, and BPF, compared to those who were nonobese (1.5, 0.4, and 0.4 µg·L^−1^ vs. 1.1, 0.3, and 0.3 µg·L^−1^ of BPA, BPS, and BPF, respectively, for obese vs. nonobese participants, respectively). However, only exposure to BPA showed a significant positive association with developing obesity; such an association was not observed with exposure to BPS or BPF. However, it should be considered that the MUC of BPS or BPF was much lower than that of BPA. Therefore, BPS or BPF might have shown a significant association among those participants if they were exposed to a similar level of BPA. In addition, the data of this study were carried out in the year 2013–2014, and the replacement of BPA with BPS and/or BPF in some products has only been applied for a couple of years; therefore, the development of obesity as a result of chronic exposure to an obesogenic environmental agent might be seen over a long timescale. Moreover, this study found an association between exposure to BPS and elevated body mass index (BMI) levels, but those who had this association did not yet develop significant clinical obesity. Therefore, exposure to BPS might promote weight gain, and significant obesity may develop over time. In another sample of NHANES (2013–2014) [31], including children and adolescents aged 6–17, results showed a significant association between exposure to BPA and BPF (MUC, 1.2 and 0.3 µg·L^−1^, respectively) with developing general obesity, but BPS (MUC, 0.3 µg·L^−1^) still did not show such a significant association. Although BPS was positively associated with elevated BMI levels and waist-to-height ratio, statistical results were nonsignificant, consistently indicating the possibility that BPS promotes weight gain. Nonetheless, exposure to BPS (MUC, 0.4 µg·L^−1^) in another NHANES sample of children and adolescents (2013–2016) [32] showed a positive association with developing obesity in a log transformation analysis. For each increase in log units in BPS, the risk of developing general, abdominal, and severe obesity increased by 16%, 13%, and 18%, respectively. In comparison, exposure to BPF (MUC, 0.2 µg·L^−1^) was positively associated with abdominal obesity and the overall BMI z-scores. Interestingly, BPA exposure in this sample did not show such results. In another US sample, including only children from the Health Outcomes and Measures of the Environment (HOME) follow-up cohort [33], exposure to BPS (MUC, 0.4 µg·L^−1^) at the age of 8 years had no significant relation to developing general obesity at the age of 8 and 12 years. However, results showed that exposure to BPS was positively associated with an increase in waist circumference at age 8. In addition, with each 10-fold increment in BPS urinary concentrations, there was a modest increase in body fat percentage at age 8 years. Nonetheless, it should be taken into account that this study used only a single urine sample to assess the exposure to BPS, which may have caused misleading results.

On the other hand, some of the reported studies showed differing results based on sample sex; Liu and colleagues [31] found that BPA and BPF were more associated with developing obesity in boys than girls. The MUC of BPA and BPF of both boys and girls did not differ significantly. Therefore, hormone differences can alter the susceptibility to adverse effects related to BP exposure. Similarly, in a Korean cross-sectional study, including more than 3000 adults aged 19 years or older from the Korean National Environmental Health Survey 2015–2017 [34], BPS exposure (MUC, 0.03 µg·L^−1^) showed a more significant association with developing obesity in males than females. Although conflicting results were reported by Gajjar et al. [33], increased waist circumference at 8 years due to exposure to BPS mostly emerged in girls but not boys. In another study, elevated BMI levels were only observed in females consuming food highly exposed to total BPs [19]. Nonetheless, future studies are highly recommended to fully understand the effect of sex differences on altering the susceptibilities to BP exposure’s adverse effects.

Lastly, it can be concluded from the results of four studies that exposure to BPS was not associated with the development of obesity. However, one of the four studies by Jacobson et al. [32] reported a positive association between BPS (MUC, 0.4 µg·L^−1^) exposure and the development of obesity in a log transformation analysis. The incidence of general obesity in this study increased by 16%. Furthermore, most studies reported that BPS exposure was related to increased BMI and/or waist circumference. These results also showed sex differences; however, the results were conflicting. In one study, the adverse effects of BPS emerged more in males than females, whereas another study reported the opposite meaning. This highly indicates the possibility of sex hormones altering the susceptibility to the adverse effects of BPS. For BPF, two of the four studies reported an association with the development of obesity at exposure levels ranging from 0.2 to 0.3 µg·L^−1^ of MUC; results also showed sex differences. Up to this point, we can conclude that BPF exposure could promote the development of obesity. However, BPS exposure may promote weight gain in the long term. Nonetheless, it must be taken into account that most of these studies were conducted in the US; therefore, other epidemiological studies in different regions are highly needed. In addition, future studies such as randomized clinical trials are recommended.

### 2.2. Incidence of Diabetes

As shown in Table 1, the association between urinary concentrations of BPS or BPF and developing diabetes has been studied in multiple studies. In a case–control study in China [35], diabetic individuals had higher BPS concentrations in their urinary samples than non-diabetic individuals. The urinary detection rates of BPS in the diabetic and nondiabetic groups were 68.1% vs. 47.8%, respectively. For BPF, however, the nondiabetic group had higher detection rates than the diabetic group (37.1% vs. 26.3% in nondiabetic vs. diabetic, respectively). At this level of exposure, BPS showed a significant positive association with the incidence of T2D. Even after further adjustments of multiple covariates, the association remained significant. A French case–cohort study also observed a strong association between urinary detection rates of BPS and the incidence of diabetes [36]. Participants were followed up for over 9 years and were nondiabetic at baseline; BPS exposure (MUC, 0.1 µg·L^−1^) was associated with the incidence of diabetes both at baseline and in the third year. Such an association emerged more in females than males, which consistently indicates the effect of sex differences in altering the adherence to the adverse effects of BP exposure. However, the sex differences were only found with exposure to BPS but not BPA. Consistently, in a Korean cross-sectional study by Moon et al. [34], exposure to BPS (MUC, 0.03 µg·L^−1^) showed a significant positive association with developing diabetes. Concentrations above 0.019 μg·L^−1^ for BPS exceeded the exposure limitation level and were divided into tertiles; each increase in BPS tertile was associated with an increase in diabetes risk by 1.5 times in males but not in females. For BPF (MUC, 0.1 µg·L^−1^), no association with developing diabetes was observed.

Furthermore, exposure to BPS may also be associated with developing gestational diabetes. In a prospective Chinese cohort [37], BPS was detected in 90% of urine samples of more than 1800 pregnant women. At this level of exposure, measured at the 13th week on average, BPS was significantly associated with an increase in fasting plasma glucose levels and 1 h postprandial glucose at the 24th to 28th weeks. More interestingly, this association was significantly observed in women carrying a female fetus, strongly indicating the effect of fetal sex in altering the susceptibility and vulnerability to BPS exposure’s adverse effects during pregnancy. However, BPS generally did not show a significant positive association with the incidence of gestational diabetes. No association was observed with increased glucose levels for BPF (detected in 94.72% of samples).

It can be concluded from what has been stated so far that BPS exposure can increase the incidence of diabetes. Results of three studies showed a positive association between BPS exposure (MUC, 0.1–0.3 µg·L^−1^) and the incidence of diabetes. Similar to obesity findings in the previous subsection, the association differed according to sex differences; however, studies showed conflicting results. For BPF exposure, only one study reported that BPF (MUC, 0.11 µg·L^−1^) was not associated with developing diabetes; unfortunately, other studies only assessed BPS exposure. Furthermore, exposure to BPS may slightly promote gestational diabetes; one study reported that BPS exposure was associated with elevated gestational blood glucose levels. However, no significant association was observed with developing gestational diabetes. Exposure to BPF also showed no association with elevated gestational blood glucose. Nonetheless, studies investigating the effects of BPS and/or BPF exposure on the incidence of diabetes are limited. Further studies are highly suggested to confirm the possible association between BPS and/or BPF exposure and the incidence of diabetes and gestational diabetes.

## 3. Exposure to BPS and/or BPF and Obesogenic Effects/Metabolic Disorders

In several *in vivo* and *in vitro* studies, the potential obesogenic effects and metabolic disturbances mediated by BPS or BPF treatments were investigated (Table 2). This section discusses the findings of these studies and explains the possible mechanisms underlying the adverse effects of BPS or BPF.

In male rats orally administered with BPS at 30, 60, and 120 mg·kg^−1^ body weight (BW) daily for 30 days, the results showed imbalances in serum glucose and lipids. Significant increases in total cholesterol, triglyceride, glycerol-free triglyceride, and low-density lipoprotein (LDL) cholesterol, with a significant decrease in high-density lipoprotein (HDL) cholesterol, were observed compared to controls [38]. In another study, the oral administration of BPS at a much lower exposure level (0.05 mg·kg^−1^ BW) over the long term showed a significant increase in body weight by almost 14% compared to controls. Elevated levels of triglycerides were also observed. Interestingly, such results were not observed in rats administered with BPA at similar conditions, indicating that BPS could be more potent in inducing obesity than BPA [39]. The oral administration of BPS in the short term at a higher dose (5 mg·kg^−1^ BW) showed similar results in female mice. After 2 weeks of administration, the female mice gained significant body weight by almost 31% ± 4% compared to controls. BPS was significantly associated with an increase in visceral fat formation. Consistently, this association emerged more in mice administered with BPS but not those with BPA. However, at a higher exposure level of BPS (50 mg·kg^−1^ BW), there were no changes in body weight compared to controls. Although this level of exposure resulted in a massive liver injury based on significant increases in serum alanine aminotransferase (ALT) and aspartate aminotransferase (AST) [40]. The promotion of visceral fat accumulation was also observed in zebrafish exposed to BPS at 1, 10, and 100 µg/L doses. Significant increases in triacylglycerol levels were observed in the BPS-exposed groups [41].

Similarly, in zebrafish exposed to BPS over the long term, significant increases in plasma triacylglycerol levels, free fatty acids, total cholesterol, and LDL cholesterol levels were observed compared to controls. Fat accumulation was also observed in liver tissues. However, in female zebrafish, only free fatty acids levels were elevated, especially at the higher exposure level (1000 µg·L^−1^), with no excessive fat accumulation observed, which raises the evidence that sex differences can modify the susceptibility to BPS adverse effects. The sex differences might be attributed to the role of endogenous estrogen signaling in females in playing a role in liver lipid homeostasis [42].

The promotion of lipid accumulation was also reported *in vitro*; in human adipose-derived stem cells (hASCs) treated with BPS or BPF at the doses 0.01, 0.1, 1, 10, and 25 μM for 14 days, results showed a dose-dependent increase in lipid accumulation [43]. In addition, other *in vitro* studies supported the evidence of BPS being more potent than BPA in promoting obesogenic effects. In murine 3T3-L1 preadipocytes, lipid accumulation and upregulation of adipogenic gene expression were observed more in BPS-treated cells than in those treated with BPA [44]. Similarly, in other murine 3T3-L1 preadipocytes exposed to BPA, BPS, or BPF for 12 days, the adipocytes were more sensitive to BPS exposure at low concentrations than BPA or BPF. BPS had the greatest impact in increasing lipid accumulation, followed by BPA, with BPF having the least impact [45]. BPF seemed to affect body weight slightly but inversely; Drobna et al. [45] performed an *in vivo* investigation and showed that male mice fed with chow exposed to BPF in different concentrations for 12 weeks had gained less body weight than controls. In addition, BPF did not promote any alterations in glucose homeostasis.

In contrast, BPS was reported to promote a significant alteration in glucose and insulin homeostasis in another study; male rats orally administered with BPS at the doses of 0.05, 0.5, and 5 mg·kg^−1^ BW for 90 days showed impairments in glucose tolerance and reduced insulin response at the highest exposure level (5 mg·kg^−1^ BW) [29]. Hyperglycemic effects were also shown in male zebrafish exposed to BPS for 28 days; a significant increase in fasting blood glucose and a decrease in insulin levels was observed in the BPS-exposed group compared to the control. Consistently, BPS was shown to impair glucose homeostasis by inducing disturbances in gluconeogenesis, glycogenolysis, and glycogen synthesis pathways [46]. Moreover, BPS exposure for 38 weeks in male rats was shown to promote significant disturbances in glucose homeostasis metabolism. Such results were not observed with BPA treatment, indicating that BPS could promote more potent hyperglycemic effects than BPA [39].

Furthermore, perinatal exposure to BPS was reported to promote or accelerate the progression of obesity in male mice offspring. Mice dams were exposed to BPS from day 7 of gestation until postnatal day 21; after 10 weeks of feeding the mice offspring with a high-fat diet (HFD), results showed a significant increase in body weight compared to mice offspring fed with HFD, but their dams were not exposed to BPS. Elevated triglycerides and total cholesterol levels were also observed. In addition, lipid accumulation showed a significant acceleration in the mice offspring’s liver tissues and epididymal white adipose tissues [47]. Consistently, perinatal exposure to BPS from day 9 of gestation until delivery increased the susceptibility to HFD-induced adipogenesis in male mice offspring. Results showed significant gonadal adipocyte hypertrophy attributed to BPS effects in upregulating expression of adipogenic genes in the enlarged gonadal adipose tissues [48].

Lastly, we can conclude from the *in vivo* and *in vitro* studies that BPS exposure could promote obesogenic effects/metabolic disorders. The administration of BPS in several studies resulted in imbalances in glucose and lipid homeostasis. An increase in fat accumulation was also reported. Interestingly, some studies showed that BPS could be more potent than BPA in prompting these adverse effects. In addition, sex differences were reported, increasing the evidence that sex differences could alter the susceptibility to the adverse effects of BPs. For BPF exposure, one study reported inverse adverse effects on body weight; rats gained less weight compared to normal rats with no alterations in glucose homeostasis. Nonetheless, further studies are suggested to confirm these findings. Furthermore, perinatal exposure to BPS was also reported to significantly increase the susceptibility to HFD-induced obesity in mice offspring.

## 4. Possible Mechanisms of Action

Various pathophysiological mechanisms could mediate the obesogenic effects/metabolic disorders of BPs; in several studies, BPA, BPS, or BPF were reported to exert obesogenic effects mainly via promoting estrogenic or androgenic activities. However, these were not the only reported mechanisms; BPs could also promote significant alterations in the gene expressions of different adipogenic-related markers. In addition, BPs were reported to promote oxidative stress and an inflammatory state. This section discusses these possible mechanisms.

Several *in vivo* and *in vitro* studies have demonstrated that BPA has adverse effects on binding to nuclear hormone receptors such as estrogen receptors (ERs), ERα and ERβ, or androgen receptors (ARs) [49,50,51,52]. ERs and ARs are considered therapeutic targets for preventing obesity and metabolic disorders due to their roles in regulating lipid accumulation and improving insulin sensitivity [53].

Similar to BPA, BPS and/or BPF were reported to promote estrogenic and androgenic activities; however, BPS only showed binding ability to ERs but not to ARs [54]. Another study reported that all three BPs (BPA, BPS, or BPF) individually showed estrogen agonist and androgen antagonist activities. The mixture of the three BPs promoted similar activities at lower concentrations compared to each BP alone [55].

In addition to estrogenic or androgenic activities, obesogenic effects of BPA and its analogs were attributed to disturbances in the upregulation of gene expressions of multiple critical adipogenesis-related markers such as PPARγ and C/EBPα [43]. PPARγ and C/EBPα, peroxisome proliferator-activated receptor gamma and CCAT/enhancer-binding protein alpha, are critical adipogenic transcription factors playing roles in adipogenesis and glucose metabolism. Both regulate each other’s expression positively and cooperate in controlling adipogenesis [56,57]. In several studies, exposure to BPA analogs, BPS or BPF, was reported to promote significant alterations in the upregulation of PPARγ and C/EBPα. In 3T3-L1 preadipocytes incubated with BPA, BPS, or BPF at 20 μM for 10 days, results showed that all three BPs promoted upregulations in the adipogenic markers PPARγ and C/EBPα [58]. Martínez et al. [27] performed a similar experiment and showed that BPS-treated preadipocytes had more significant upregulation in PPARγ and C/EBPα, followed by BPF, and then by BPA. It was indicated that BPS and BPF might be more potent in promoting obesogenic effects than BPA. Similar results were reported in 3T3-L1 preadipocytes incubated with BPA or BPS at 50 μM for 8 days; BPS-treated cells showed higher lipid accumulation attributed to significant upregulation of PPARγ and C/EBPα compared to BPA-treated cells [59]. In human preadipocytes treated with BPS, consistent results were reported according to the upregulation of PPARγ [59]. Consistently, in hASCs incubated with BPS or BPF at 10 and 25 μM for 14 days, results showed a significant dose–response alteration in PPARγ and C/EBPα expression [43].

Disturbances in other adipogenic-related markers, such as *lipoprotein lipase* (LPL) and *fatty acid-binding protein 4* (FABP4), were also reported [27,43,59]. LPL is an extracellular enzyme playing a critical role in the hydrolysis of triglycerides to fatty acids and glycerol [60], and FABP4 is a transport protein playing a key role in developing insulin resistance and atherosclerosis [61]. It was reported that the intracellular lipid accumulation after BPS or BPF incubation was also attributed to upregulations in FABP4 and LPL [43,59]. Similar results were shown in other studies; in human preadipocytes incubated with BPS at 25 μM BPS for 12 days, lipid accumulation was attributed to upregulations in the expressions of FABP4 and LPL [62]. In 3T3-L1 cells incubated with BPA, BPS, or BPF at doses ranging from 1 to 40 μM for 6 days, the results showed that both BPS and BPF similarly promoted lipid accumulation to BPA; these results were attributed to upregulations in FABP4 and LPL [28].

On the other hand, the development of obesity and metabolic disorders has been demonstrated to be associated with oxidative stress [63]. Indeed, a recent study showed that insulin resistance, the main factor attributed to the pathogenesis of obesity and diabetes, was the strongest metabolic component associated with the presence of oxidative stress [64]. Oxidative stress is defined by an imbalance between the production of reactive oxygen species, known as free radicals, and the antioxidants in the body [65]. Several studies have demonstrated that exposure to BPA induces oxidative stress [66,67,68]. Indeed, BPA analogs, BPS and BPF, have also been shown to promote disturbances in the endogenous antioxidant system in several studies [58,69,70,71]. In RWPE-1 cells, the incubation with BPS or BPF at doses ranging from 0 to 600 μM for 24 h showed imbalances in the levels of oxidative stress markers such as superoxide dismutase, glutathione peroxidase, and glutathione reductase activities, as well as in the levels of glutathione and the total antioxidant capacity compared to the nonexposed groups [72]. Similarly, in hepatocytes exposed to BPS at doses ranging from zero to 500 μM for 24 h, results showed imbalances in the superoxide dismutase, catalase, and glutathione peroxidase levels [73]. Moreover, the MUC of BPF (detected in ≥85% of urinary samples) for adult individuals was significantly associated with imbalances in oxidative stress markers [74]. Furthermore, perinatal exposure to BPF also resulted in significant imbalances in the catalase activity of offspring, indicating the adverse effects of BPs exposure on early life [75].

Furthermore, exposure to BPs could also promote significant disturbances in the endogenous inflammatory response [76,77,78,79]. The body’s inflammatory responses play key roles in developing obesity and metabolic disorders [80]. These responses are upregulated by proinflammatory cytokines, which, when overexpressed, promote significant alterations. For instance, the overexpression of tumor necrosis factor-alpha (TNF-α) and interleukins (IL) cytokines, that play major roles in metabolic pathways, can promote the pathogenesis of diabetes. The overexpression of TNF-α can result in dyslipidemia and insulin resistance [81], and overexpression of IL-1β can destroy the pancreatic β-cells [82,83]. Elevated TNF-α and IL were reported after BP exposure in multiple studies; in zebrafish treated with BPS or BPF at doses ranging from 1 to 1000 μg·L^−1^ BW for 14 days, results showed that both BPS and BPF, individually or combined, promoted a significant increase in TNF-α and IL-1β expression [76]. Similarly, in mice orally administered with BPA, BPS, or BPF at 0.5, 5, and 50 μg·kg^−1^ BW doses for 5 weeks, results showed impaired oral tolerance attributed to an increase in TNF-α expression. However, these results were only observed in BPF-exposed mice [77]. Consistently, in an *in vitro* study using RAW264.7 cells, BPF exposure at doses ranging from 0 to 20 mM for 24 h resulted in a dose-dependent increase in the expression of different proinflammatory cytokines, including TNF-α, IL-6, and IL-1β [78]. In another *in vitro* study, naïve mice T cells were treated with BPA, BPS, or BPF at doses ranging from 0.05 to 50,000 nM; results showed that both BPS and BPF, individually, but not BPA, promoted a significant increase in IL-17 at the lowest levels, indicating the possible adverse effects of BPs at a low and environmentally relevant concentration [79].

## 5. Future Work

According to relevant studies, BPS and/or BPF could promote the development of obesity and diabetes, with BPS potentially being more potent. However, according to our literature review, we found a study reporting unexpected and conflicting results; in male mice exposed orally to BPS over the long term, hypoglycemic effects were observed, and results were attributed to an increase in insulin sensitivity and a decrease in gluconeogenesis mediated by the inhibition of thyroid hormone-signaling responses [84]. The number of studies reporting the adverse effects of BPs, in general, is increasing. Nevertheless, future studies are highly needed to confirm or support the evidence regarding the obesogenic effects/metabolic disorders of BPS or BPF as a substitute for BPA.

Furthermore, most studies reporting an association of urinary concentrations of BPS or BPF with obesity or diabetes were carried out in the US; therefore, other studies in different countries and populations should be conducted in the future. BPs, in general, are rapidly metabolized in the human body [21,85]. Thus, urine samples cannot reflect the exposure level in the long term. On the other hand, sex differences were reported to modify the susceptibility to the adverse effects of BPs. Some studies reported obesogenic effects and metabolic disturbances emerging more in males than females, while others reported the opposite. Studies reporting females being less susceptible to obesogenic effects/metabolic disorders of BPs indicated that endogenous estrogen signaling in females might play a role in the homeostasis of lipid metabolism. A study reported that a phytoestrogen-based diet could prevent BPS effects in inducing diabetes, but results differed according to sex [86]. Therefore, future investigations are highly needed to fully understand or uncover differences in the mode of action of BPs according to sex.

## 6. Conclusions

Exposure to BPS or BPF could promote the development of obesity and diabetes, with BPS having the most impact. On the basis of median urinary concentrations, BPS exposure has been linked to elevated BMI levels and waist circumference. However, for BPF exposure, some studies showed an association with developing obesity, while others showed null results. Future studies in different countries and populations, such as prospective cohorts and controlled trials, are recommended to confirm these results. Furthermore, the findings of experimental studies showed that BPS could promote significant disturbances in lipid and glucose metabolism and increase fat accumulation. The possible mechanisms of action are attributed to estrogenic or androgenic activities, promoting alterations in the gene expression of adipogenic-related markers, and inducing oxidative stress and an inflammatory state. Additionally, the potency of BPS, BPF, or total BPs may differ according to sex. Some studies showed that the obesogenic effects of BPs emerged more in males than females, while others showed the opposite. Therefore, future studies are highly needed to fully uncover or understand the possible mode of action underlying sex differences in the susceptibility to BPs-induced obesogenic and metabolic disorders.

## Figures and Tables

**Table 1:** Summary of studies reporting the association between BPS and BPF exposure and the incidence of obesity and diabetes in human populations.

Study Design/Follow-Up	BPs Exposure Level MUC (µg·L^−1^)/DR (%)	Main Outcomes	References
Participant Characteristics			
Cross-sectional/- *n* = 1521 adults (age ≥ 20 years)	BPS: 0.4/- BPF: 0.4/-	Comparing the highest with the lowest quartile of exposure level: No significant association with general obesity; BPS [OR 1.22, 95% CI = 0.81–1.83, *p* = 0.30] BPF [OR 1.02, 95% CI = 0.70–1.47, *p* = 0.81]	Liu et al. [30] (2017, United States)
Cross-sectional/- *n* = 745 children (age 6–17 years)	BPS: 0.3/- BPF: 0.3/-	Comparing the highest with the lowest quartile of exposure level: ↑ General obesity risk with BPF; [OR 1.54, 95% CI = 1.02–2.32, *p* = 0.05] (stronger in boys than in girls), boys [OR 3.35, 95% CI = 2.02–5.53, *p* < 0.001] girls [OR 0.55, 95% CI = 0.25–1.25, *p* = 0.13] BPS had no significant association with obesity risk	Liu et al. [31] (2019, United States)
Cross-sectional/- *n* = 1831 children and adolescents (age 6–19 years)	BPS: 0.4/87.8 BPF: 0.2/55.2	Each increment in log units of BPS UC: ↑ Risk of general, abdominal, and severe obesity by 16%, 13%, and 18%, respectively; general obesity [OR 1.16, 95% CI = 1.02–1.32] abdominal obesity [OR 1.13, 95% CI = 1.02–1.27] severe obesity [OR 1.18, 95% CI = 1.03–1.35] BPF exposure: ↑ Abdominal obesity and overall BMI z-scores abdominal obesity [OR 1.29, 95% CI =1.01–1.64] BMI z-score [β = 5 0.10, 95% CI =0.01-0.20]	Jacobson et al. [32] (2019, United States)
Cross-sectional/- *n* = 212 and 181 children (age 8 and 12 years, respectively)	BPS: 0.4/- at age 8 years	Each 10-fold increment in BPS UC at age 8 years: ↑ Body fat (%) modestly; [β = 1.1, 95% CI = −0.6–2.7] Girls at age 8 years: ↑ Waist circumference; [β = 1.4, 95% CI = −1.6–4.5]	Gajjar et al. [33] (2022, United States)
Cross-sectional/- *n* = 3777 adults (age ≥ 19 years)	BPS: 0.03/55.2 BPF: 0.11/44.1	Comparing the highest with the lowest tertiles of BPS exposure: ↑ Obesity risk in males; [OR 2.12, 95% CI = 1.07–4.21, *p* < 0.032] Each increment in BPS tertiles: ↑ Diabetes risk by 1.5 times in males; [OR 1.50, 95% CI =1.11–2.01, *p* = 0.009] BPF had no association with the diabetes risk	Moon et al. [34] (2022, Korea)
Case–control/- *n* = 251 T2D adults (age ≥ 19 years)	BPS: -/68.1 BPF: -/26.3	Each increment in log units of BPS UC: ↑ Risk of T2D; [OR 1.46, 95% CI = 1.22–1.74, *p* < 0.001]	Duan et al. [35] (2018, China)
Case–cohort/9 years *n* = 755 adults (age 30–65 years)	BPS: 0.18/- BPF: -/-	With BPS exposure: ↑ T2D risk; (stronger in females than in males), females [HR 4.23, 95% CI = 1.69–10.63] vs. males [HR 1.76, 95% CI = 0.93–3.33] (*p* = 0:09)	Rancière et al. [36] (2019, France)
Prospective cohort/ *n* = 1841 pregnant women (gestational age < 16 weeks)	BPS: 0.3/90.06 BPF: 1.74/94.72	With the increase in BPS detection rate: ↑ FPG + 1 h PPG; stronger in women carrying a female fetus [*p* < 0.05 for FPG and < 0.01 for 1 h PPG]	Zhang et al. [37] (2019, China)

Abbreviations: BPs: bisphenols; BPS; bisphenol S; BPF: bisphenol F; MUC: median urinary concentrations; DR: detection rate; T_2_D: type 2 diabetes; FPG: fasting plasma glucose; PPG: postprandial glucose; (↑): increase; OR: odds ratio; HR: hazard ratio; CI: confidence interval.

**Table 2:** Summary of *in vivo* and *in vitro* studies reporting the obesogenic and diabetic effects of BPS and BPF exposure.

Model System	BPs Exposure System/	Main Outcomes	Reference
	Dose/Period		
Male rats	Oral administration by gavage BPS (30, 60, and 120 mg·kg^−1^ BW)/daily for 30 days	↑ Serum glucose ↑ Total cholesterol, triglyceride, glycerol-free triglyceride, and LDL ↓ HDL	[38]
Male rats	Administered with drinking water BPA or BPS (0.05 mg·kg^−1^ BW)/38 weeks	↑ BW by 14% after 32 weeks ↑ Triglycerides ↑ Blood glucose No effects with BPA	[39]
Female mice	Oral administration by gavage BPS (0.5, 5, and 50 mg·kg^−1^ BW)/daily for 2 weeks	BPS at 5 mg·kg^−1^ BW dose: ↑ BW by 31% ± 4% ↑ Visceral fat formation (emerged more with BPS exposure but not with BPA) BPS at 50 mg·kg^−1^ BW dose: ↑ ALT and AST	[40]
Zebrafish	Diluted in water (refreshed daily) BPS (1, 10, and 100 µg·L^−1^)/15 days	↑ Visceral fat accumulation ↑ Triacylglycerol	[41]
Zebrafish	Diluted in water (refreshed daily) BPS (1, 10, 100, and 1000 µg·L^−1^)/120 days	In males: ↑ FFA, triacylglycerol, total cholesterol, and LDL ↑ Fat accumulation in the liver In females: ↑ FFA emerged with (1000 µg·L^−1^)	[42]
Adipose hASCs	BPs solution added to media BPS or BPF (0.01, 0.1, 1, 10, and 25 μM)/14 days	↑ Lipid accumulation ↑ Adipogenesis	[43]
Murine 3T3-L1 preadipocytes	BPs solution added to media (replaced and refreshed every 2 days) BPA or BPS (0.01–50 μM)/6 days	↑ Lipid accumulation ↑ Upregulation of adipogenic genes expression (emerged more in BPS than BPA)	[44]
Murine 3T3-L1 preadipocytes	BPs solution added to media (replaced and refreshed every other day) BPA, BPS, or BPF (10 nM)/12 days	↑ Lipid accumulation with BPS, followed by BPA BPF had the least impact	[45]
Male mice	Administered with chow BPF (0, 0.5, 5, and 50 mg·kg^−1^ chow ≈ 0.044, 0.44, and 4.4 mg·kg^−1^ BW)/daily for 12 weeks	Mice gained less weight than controls No effects on glucose levels or glucose tolerance	[45]
Male rats	Oral administration by gavage BPS (0.05, 0.5, and 5 mg·kg^−1^ BW)/daily for 90 days	With median and high doses: ↑ Blood glucose ↓ Insulin response ↑ Disturbances in glycolysis and gluconeogenesis	[29]
Male zebrafish	Diluted in water (refreshed daily) BPS (1 and 10 μg·L^−1^)/28 days	↑ FBG ↓ Insulin levels	[46]
HFD-induced male mice	Oral administration by gavage to dams BPS (0.1 mg·kg^−1^ BW)/daily from gestational day 7 to postnatal day 21	Compared to HFD-induced offspring of dams not exposed to BPS: ↑ BW ↑ Triglycerides and total cholesterol ↑ Lipid accumulation in liver tissues and epididymal white adipose tissues	[47]
HFD-induced male mice	Administered with drinking water of dams BPS (0.05, 0.5, 5, and 50 mg·kg^−1^ BW)/daily from gestation day 9 until delivery	↑ Adipocytes size in gonadal white adipose tissue of offspring (gonadal adipocyte hypertrophy)	[48]

Abbreviations: BPs: bisphenols; BPA: bisphenol A; BPS; bisphenol S; BPF: bisphenol F; BW: body weight; LDL: low-density lipoprotein; HDL: high-density lipoprotein; FFA: free fatty acids; HFD: high-fat diet; hASCs, human adipose-derived stem cells; ALT: alanine aminotransferase; AST: aspartate aminotransferase; FBG: fasting blood glucose; (↑): increase; (↓): decrease.

## Data Availability

Data are contained within the article.
